# Radiation-Associated Breast Angiosarcoma

**DOI:** 10.7759/cureus.46673

**Published:** 2023-10-08

**Authors:** Ryusei Yoshino, Nana Yoshida, Nanami Ujiie, Akane Ito, Masaki Nakatsubo, Yuki Kamikokura, Masahiro Kitada

**Affiliations:** 1 Thoracic Surgery and Breast Surgery, Asahikawa Medical University Hospital, Asahikawa, JPN; 2 Diagnostic Pathology, Asahikawa Medical University Hospital, Asahikawa, JPN

**Keywords:** skin biopsy, radiation therapy, breast-conserving surgery, purpura, radiation-associated breast angiosarcoma

## Abstract

Radiation-associated breast angiosarcomas are rare following breast-conserving surgery. These angiosarcomas are considered adverse events associated with radiation therapy and are characterized by a high risk of both local and distant recurrence, even after complete resection. Despite this, there is currently no established standard treatment for them. The patient was a 70-year-old woman who had breast-conserving surgery for right breast cancer 10 years before presentation. She was followed up for 10 years after receiving 50 Gy of residual breast irradiation and an aromatase inhibitor for 5 years. During follow-up, a painless purplish area with induration, measuring 10 cm by 5.4 cm, was noted on the right nipple. A skin biopsy confirmed hemangiosarcoma. Treatment included surgery with a 2.0 cm margin from the area, followed by skin excision and total mastectomy. A final diagnosis of radiation-associated breast angiosarcoma was made. Radiation-associated breast angiosarcoma is a rare disease with a poor prognosis that lacks standard treatment. An aggressive skin biopsy should be considered when skin findings such as purpura are seen after breast cancer radiotherapy, as in this case.

## Introduction

Breast-conserving surgery followed by radiotherapy is the current standard treatment for early-stage breast cancer. However, radiation therapy after breast-conserving surgery has recently been reported to cause angiosarcoma; thus, the concept of radiation-associated breast angiosarcoma has been proposed [1]. The details of the pathogenesis of radiation-induced angiosarcoma have only been reported in a limited number of cases, and the treatment of angiosarcoma has not yet been established.

Angiosarcomas are commonly said to occur on the face and scalp of the elderly due to trauma to the head or other external stimuli, but they rarely occur secondary to radiation therapy. Radiation-associated breast angiosarcoma is a rare disease with a reported incidence of less than 0.05% among malignant breast tumors [2]. Clinically, radiation-associated breast angiosarcomas present with swelling, purpura, erythema, nodules, ulceration, and skin thickening. Furthermore, this condition has a poor prognosis, and the recommended treatment is complete surgical resection; however, there are no clear criteria regarding the extent of such a resection [3].

We report the case of radiation-associated breast angiosarcoma diagnosed by early skin biopsy 10 years after the completion of postoperative radiotherapy for breast cancer.

## Case presentation

**Figure 1:**
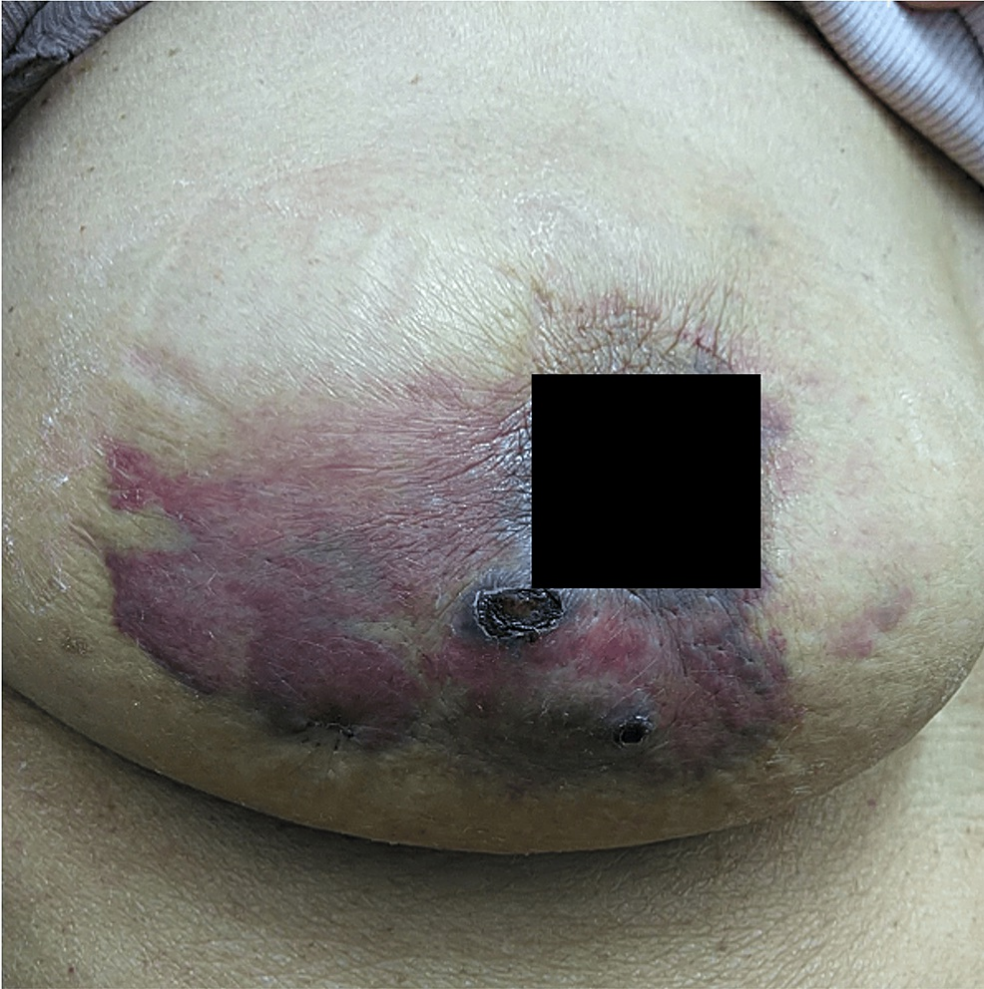
Physical examination A 10 cm × 5.4 cm area of painless purpura around the right nipple with some induration was observed.

The patient is a 70-year-old woman who underwent a partial mastectomy and sentinel lymphadenectomy for right breast cancer 10 years ago, followed by 50 Gy radiation and oral hormone therapy for five years. Thereafter, the patient attended annual follow-up examinations for 10 years. During a periodic check-up in the 10th year, a partially indurated, painless purpura with an area of 10 cm × 5.4 cm was observed in an external inferior direction from the right nipple (Figure 1). No enlarged supraclavicular, subclavian, axillary lymph nodes, edema, or conjunctival icterus was observed. There were no differences between the right and left respiratory sounds, and the heart sounds were also normal. Blood samples were collected, and the test results revealed that the tumor markers (CEA and CA15-3) were within the standard range, and there were no other abnormal values observed in the general complete blood count or biochemical tests. Electrocardiography (ECG) showed atrial fibrillation, which was originally noted; however, the heart rate was within the normal range. Respiratory function tests revealed no obstructive or restrictive ventilatory impairments.

**Figure 2:**
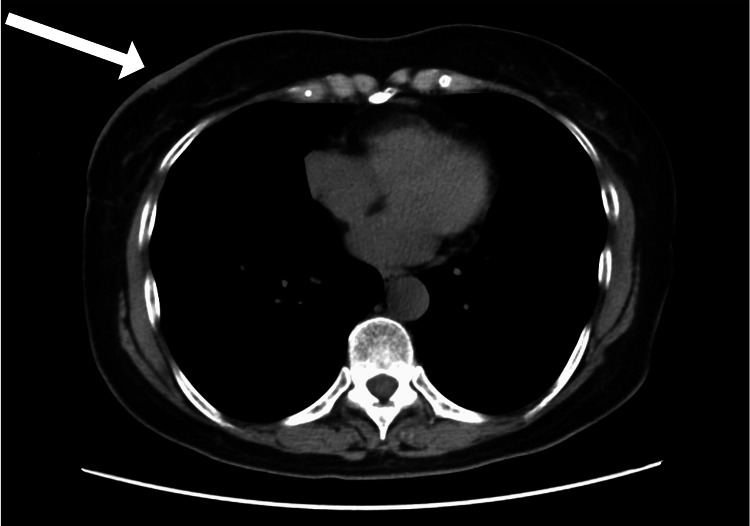
Chest computed tomography (CT) findings The section showed thickening of the skin in the right breast, but no evident mass lesion was found in the mammary gland.

**Figure 3:**
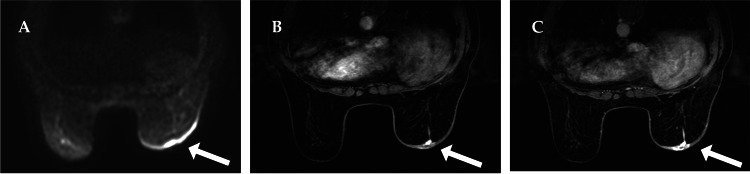
Contrast-enhanced magnetic resonance imaging (MRI) of the breast findings A: Right breast skin thickening with high signal along the thickened area (STIR/DWI). B: In the dynamic study, there was a strong enhancement effect in the subcutaneous area in the form of dots and an enhancement effect predominantly at the limbus on the skin surface (early phase). C: Prolonged enhancing effect (late phase).

A mammogram revealed an indistinct mass shadow in the right nipple and thickening of the nipple wall. Breast ultrasonography revealed no apparent neoplastic lesions in the mammary glands, and no enlarged axillary lymph nodes were observed. A computed tomography (CT) scan of the thorax and abdomen showed no evidence of recurrence or metastasis; however, thickening of the skin on the right breast was observed (Figure 2). No apparent lesions were observed in the mammary glands. A magnetic resonance imaging (MRI) of the breast showed no mass lesions, and a short tau inversion recovery (STIR) and diffusion weighted image (DWI) showed a high signal along the area of right breast skin thickening. Dynamic MRI showed a strong enhancement effect in the early phase, with strong subcutaneous point-like and predominant enhancement effects on the skin surface at the limbus; a prolonged enhancement effect was observed toward the late phase (Figure 3). There was no obvious evidence of recurrence or metastasis on the CT of the chest or abdomen, and positron emission tomography (PET) was not performed.

**Figure 4:**
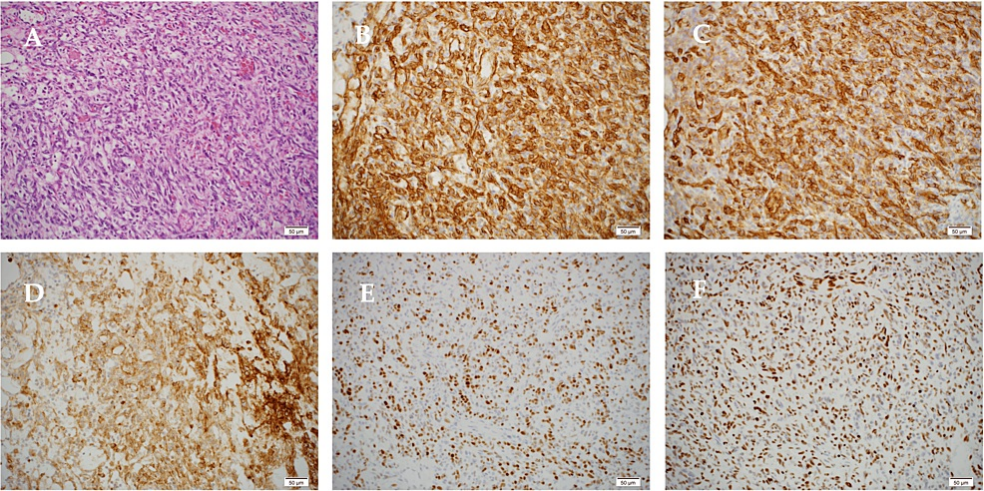
Histopathological findings A: Atypical cells with irregularly shaped enlarged nuclei stained with chromatin were observed from the dermis to the adipose tissue using hematoxylin-eosin (HE) staining. Scale bar: 50 μm. B: Aberrant cells positive for CD31. C: Aberrant cells positive for CD34. D: Aberrant cells positive for D2-40 E: Diffusely positive c-Myc. F: partial loss of H3K27me3 expression.

**Figure 5:**
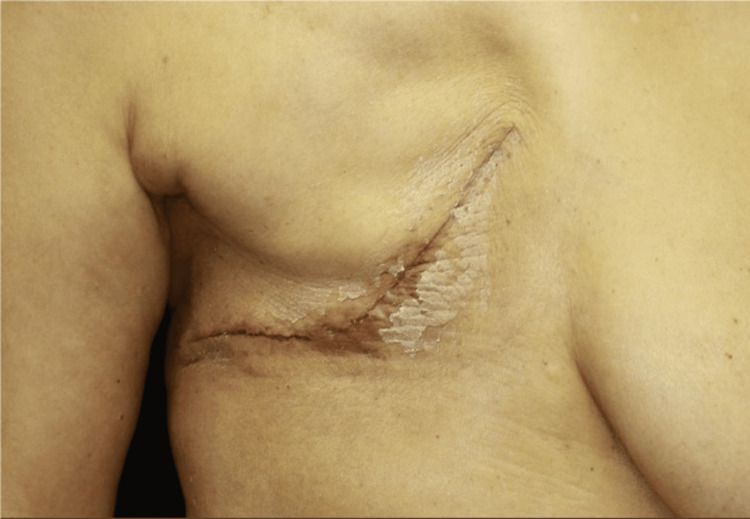
Physical examination Skin after a partial mastectomy. No additional skin grafting was performed.

A skin biopsy of the purple lesion on the right breast was performed in the dermatology department. Histopathological examination revealed numerous small and large voids in the upper and lower dermis, and marked extravascular erythrocyte extravasation was also observed. The lumens of the voids were partially lined with spindle-shaped cells with large nuclei, which formed incomplete vascular vessels. Immunohistochemistry results were positive for CD31, CD34, and D2-40 and negative for HHV-8. Based on the biopsy results, the patient was preoperatively suspected of having an angiosarcoma of the right breast. Radiation-associated breast angiosarcoma was also suspected because of the previous radiotherapy. Therefore, a skin excision and right mastectomy were performed with a margin of 2.0 cm from the purpura. Two-stage skin grafting was considered because of the large extent of the excision. Histopathological examination revealed atypical cells with irregularly shaped, enlarged nuclei stained with chromatin darkening from the dermis to the adipose tissue, which proliferated between the collagen fibers and adipose tissue, forming irregularly branched and anastomosed vascular cavities. The atypical spindle-shaped cells proliferated intricately in the bundles. Immunohistochemistry showed that the atypical cells were positive for CD31, CD34, and D2-40 and negative for AE1/AE3. In addition, c-Myc was diffusely positive, and H3K27me3 expression was partially absent (Figure 4). Based on histological findings, the tumor diameter was 18 × 15 mm, and the FNCLCC histological grade was 2 (tumor differentiation, score 2; mitotic count, score 3; tumor necrosis, score 0; total score, 5). Resection margins were negative. In instances where the margins were positive, additional skin excision and grafting were considered; however, these were not performed (Figure 5). The patient was administered adjuvant paclitaxel postoperatively.

## Discussion

Radiation-associated breast angiosarcoma can occur several years after radiation therapy, with some reported cases occurring decades later [4]. The prognosis is generally poor, with a five-year recurrence-free survival rate of approximately 40% and an overall five-year survival rate of 10%-54% [5]. However, it has been reported that a tumor size greater than 5 cm, advanced age, multiple skin lesions, and the histological grade of the tumor [1,2] are associated with a poor prognosis. In addition, radiation-associated breast angiosarcoma not only frequently recurs locally after resection but may also cause distant metastasis to the contralateral breast, lymph nodes, or lungs, which is another factor contributing to the poor prognosis [2]. Therefore, a skin or incisional biopsy should be performed as soon as radiation-associated breast angiosarcoma is suspected [1]. This finding supports the notion that the aspiration cytology and needle biopsy results are insufficient. Salminen et al. stated that it is important to perform multiple punch biopsies of the affected breast before surgery to determine the extent of the lesion [3]. Survival rates for positive R1 and R2 resection margins are significantly lower than those for R0 resection margins [1], while a high recurrence rate of 54%-92% has been reported, even in cases of negative R0 margins. Gutkin et al. [6] stated that a margin of at least 5 mm is necessary; however, there are various reports on the need for a margin of at least 5 mm, including in total or partial mastectomies [7]. However, there have been various reports, and no definite opinions have been obtained.

In this case, 10 years after surgery, the patient complained of right breast purpura during a routine examination. The patient was referred to the dermatology department on the same day for a skin biopsy instead of a cytology or core needle biopsy, which led to an early diagnosis. Since radiation therapy is now the mainstay of breast-conserving surgery [1], it is important to consider radiation-associated breast angiosarcoma during the postoperative course.

Immunohistochemistry is useful for the diagnosis of radiation-associated breast angiosarcoma. Mentzel et al. [7] reported that radiation-associated breast angiosarcoma is closely correlated with MYC amplification, c-Myc expression, and loss of H3K27me3. MYC amplification is considered a marker of poor prognosis and has been identified in 55% of primary angiosarcomas and up to 100% of secondary angiosarcomas. Among these, MYC amplification is a promising marker in radiation-associated breast angiosarcoma. H3K27me3 is an epigenomic chemical marker that is added to proteins called histones. Histones form chromatin, which plays an important role in the regulation of gene expression [8]. H3K27me3 is a histone in which a methyl group is added to the specific amino acid lysine 27 of histone H3. Generally, when H3K27me3 is present at a particular locus, its expression is suppressed [9,10]. In the last few years, the loss of H3K27me3 has been reported in various epithelial, neural, melanocytic, and mesenchymal neoplasms and has been a very useful tool in the diagnosis of radiation-associated breast angiosarcoma [7]. In this case, c-Myc was diffusely positive, and H3K27me3 expression was partially absent, which led to the diagnosis. However, these studies are still in progress, and further studies, including other markers, are required.

Although there is no established evidence-based standard of care for adjuvant therapy, chemotherapy based on doxorubicin, paclitaxel, docetaxel, and radiation therapy are commonly used; however, there is still room for debate [2]. Paclitaxel is commonly used to treat unresectable and metastatic angiosarcoma, and pazopanib, a tyrosine kinase inhibitor, and bevacizumab, an anti-VEGF antibody, are considered second-line therapies [11]. Paclitaxel is considered very effective against angiosarcoma [12]. In other studies, the use of imatinib, a c-KIT inhibitor, has also been reported [2]. This patient is currently receiving postoperative adjuvant therapy with paclitaxel, which should be administered with careful follow-up, given the high recurrence rate.

## Conclusions

Radiation-associated breast angiosarcoma has a poor prognosis, and a skin biopsy should be performed at an early stage if suspected. Immunohistochemistry is useful for diagnosing radiation-associated breast angiosarcoma. The findings of the present case suggest that radiation-associated breast angiosarcoma should be aggressively considered in patients who have undergone prior radiotherapy for breast cancer based on skin findings that occur several years to decades later.

## References

[1] Mergancová J, Lierová A, Coufal O (2022). Radiation-associated angiosarcoma of the breast: An international multicenter analysis. Surg Oncol.

[2] Bonito FJ, de Almeida Cerejeira D, Dahlstedt-Ferreira C, Oliveira Coelho H, Rosas R (2020). Radiation-induced angiosarcoma of the breast: A review. Breast J.

[3] Salminen SH, Sampo MM, Böhling TO, Salo J, Tarkkanen M, Blomqvist CP, Hukkinen K (2022). Radiation-associated angiosarcoma of the breast: analysis of diagnostic tools in a registry-based population. Acta Radiol.

[4] Salminen SH, Sampo MM, Böhling TO, Salo J, Tarkkanen M, Blomqvist CP, Hukkinen K (2022). Radiation-associated angiosarcoma of the breast: analysis of diagnostic tools in a registry-based population. Acta Radiol.

[5] Nomoto Y, Kijima Y, Shinden Y (2018). Two cases of radiation-associated angiosarcoma of the breast. Surg Case Rep.

[6] Gutkin PM, Ganjoo KN, Lohman M (2020). Angiosarcoma of the breast: management and outcomes. Am J Clin Oncol.

[7] Mentzel T, Kiss K (2018). Reduced H3K27me3 expression in radiation-associated angiosarcoma of the breast. Virchows Arch.

[8] Sheu TG, Hunt KK, Middleton LP (2021). MYC and NOTCH1-positive postradiation cutaneous angiosarcoma of the breast. Breast J.

[9] Panse G, Mito JK, Ingram DR (2021). Radiation-associated sarcomas other than malignant peripheral nerve sheath tumours demonstrate loss of histone H3K27 trimethylation(†). Histopathology.

[10] Kuba MG, Xu B, D'Angelo SP (2021). The impact of MYC gene amplification on the clinicopathological features and prognosis of radiation-associated angiosarcomas of the breast. Histopathology.

[11] Shiraki E, Kang Y, Shibayama T, Tsuyuki S (2020). Two cases of breast angiosarcoma after breast conserving surgery. Surg Case Rep.

[12] Kokkali S, Stravodimou A, Duran-Moreno J, Koufopoulos N, Voutsadakis IA, Digklia A (2021). Chemotherapy and targeted treatments of breast sarcoma by histologic subtype. Expert Rev Anticancer Ther.

